# Clinical factors and pre-surgical depression scores predict pain intensity in cardiac surgery patients

**DOI:** 10.1186/s12871-022-01740-3

**Published:** 2022-07-04

**Authors:** Jacob Gohari, Liza Grosman-Rimon, Mattan Arazi, Noa Caspi-Avissar, Dina Granot, Sagi Gleitman, Jawdat Badarny, Alla Lubovich, Doron Sudarsky, Jordan Rimon, Shemy Carasso, Edo Y. Birati, Erez Kachel

**Affiliations:** 1 The Ruth and Bruce Rappaport Faculty of Medicine, Technion-Israel Institute of Technology, Haifa, Israel; 2 Creedmoor Psychiatric Center, Queens Village, New York City, NY USA; 3 The Lydia and Carol Kittner, Lea and Benjamin Davidai Division of Cardiovascular Medicine and Surgery, Padeh Poriya Medical Center, Tiberias, Israel; 4 The Academic College at Wingate, Wingate Institute, Netanya, Israel; 5 Department of Cardiac Surgery, Leviev HeartCenter, Sheba Medical Center, Tel Hashomer, Israel; 6 The Azrieli Faculty of Medicine, Bar-Ilan University, Zefat, Israel; 7 Faculty of Health, York University, Toronto, Canada

**Keywords:** Cardiac Surgery, Pain, Depression

## Abstract

**Background:**

Severe pain is prevalent in cardiac surgery patients and can increase cardiac complications, morbidity and mortality. The objectives of the study were to assess perioperative pain intensity and to assess predictors of pain post-cardiac surgery, including clinical characteristics and depression.

**Methods:**

A total of 98 cardiac surgery patients were included in the study. Pain intensity was assessed using a Numerical Rating System. Pain was measured one day pre-operatively and recorded daily from Post-operative Day 2 to Day 7. Clinical data were recorded and depression scores were assessed using the Center for Epidemiological Study of Depression (CES-D).

**Results:**

Pain intensity increased significantly during hospitalization from pre-operative levels, surging at 2 days post-operatively. Predictors of high pain intensity were high pre-operative CES-D scores, female gender, cardiac function, smoking and high body mass index (BMI). Significantly higher pre-operative CES-D scores were found in patients with severe pain compared to patients with no pain to moderate pain (18.23 ± 1.80 vs 12.84 ± 1.22, *p* = 0.01 pre-operatively). Patients with severe pain (NRS 7–10) had significantly higher levels of white blood cells (WBC) compared to patients with no pain-moderate pain (NRS 0–6), (*p* = 0.01). However, CES-D scores were only weakly correlated maximum WBC levels perioperatively.

**Conclusion:**

Pain intensity significantly increased following surgery, and was associated with depressive symptoms, female sex, cardiac function, BMI, and smoking. These factors may serve as a basis for identification and intervention to help prevent the transition from acute pain to chronic pain.

## Background

Depression post- cardiac surgery is highly prevalent [1], and is associated with significant mortality [2, 3]. Moreover, an increase in depressive symptoms after cardiac surgery is also associated with the occurrence of new cardiac complications [4]. Accumulating evidence has found that depression is associated with somatic symptoms, especially with pain severity [5]. Interestingly, up to two-thirds of patients with depression also had moderate to severe pain post-cardiac surgery [6]. Moreover, one-third of the patients who underwent cardiac surgery noted persistent or worsening depressive symptoms at post-discharge, with over two-thirds of these patients having pain interfering with daily living [7]. Post cardiac surgery pain is reported in up to 85% of patients, with moderate to severe pain occurring usually in the first 2–4 days post-operatively [8]. Moreover, studies have shown that 39% of patients post-cardiac surgery have persisting, chronic pain over 2 years post-operation [9].

There is increasing evidence that pain and depression share common mechanisms. Recent studies have found similarities between neuroplasticity changes induced by pain and by depression [10, 11]. In particular, the same brain regions, the prefrontal cortex, hippocampus, amygdala, anterior cingulate, and insular cortex are both implicated in depression and pain [12, 13].

Perioperative pain is also a major risk factor which can lead to the development of chronic pain, increased cardiac complications, increased morbidity and increased mortality [14–17].

Current data is limited on the coexistence of both pain and depressive symptoms in cardiac surgery patients [6, 7]. Currently, assessment of predictor of pain intensity did not include pre- and post-depressive symptoms in addition to other clinical and demographic characteristics.

However, whether pre- and post-operative depression is associated with perioperative pain in cardiac surgery patients is not well studied.

The objectives of the current study are to assess the peri-operative pain intensity, and to determine whether perioperative depression scores and clinical characteristics are predictive of post-surgical pain intensity.

## Methods

### Study design

A prospective study was conducted to investigate the relationship between reported pain scores and depressive symptoms throughout the perioperative period. Pain was measured one day pre-operatively and recorded daily from Post-operative Day 2 to Day 7.using a Numerical Rating System throughout hospitalization. Depression scores were assessed using the Center for Epidemiological Study of Depression (CES-D) at pre-operative, during hospitalization, and up to 10 days post-discharge. The study was approved by the institutional review board. Signed informed consents were obtained from all study participants.

### Patient cohort

Adult patients undergoing cardiac surgery (valvular surgery or coronary artery bypass grafting) at the Baruch Padeh Poriya Medical Center from March 2018 to June 2020 were included in this study.

*Inclusion Criteria:* 1) patients eligible for cardiac surgery (valve surgery or coronary artery bypass grafting (CABG) 2) patients ages 20 and older.

*Exclusion Criteria:* 1) Patients under the age of 20; 2) patients with end-stage renal disease requiring dialysis; 3) presence of peripheral neuropathy; Glasgow Coma Scale score < 13) patient on chronic pain medication; and 5) patients with chronic inflammatory disease were excluded from the study.

### Procedures

Clinical and surgical data were recorded from the electronic medical records. The following variables were recorded: Age, BMI, left ventricle ejection fraction (EF) less than 50%, white blood cell count, Working status, Smoking status, Diabetes, Residence, Education level, Marital status, Numeric Pain Rating, and CES-D depression scores.

#### Assessment of pain

Pain was measured using a Numerical Rating System (NRS), which is an 11 point interval scale evaluating pain from 0-to-10, with 0 characterizing no pain and 10 characterizing maximum pain [18, 19]. Pain severity was divided into 2 categories based on the NRS scores: no pain-moderate pain (score of 0–6), and severe pain (score of 7–10), [19] since severe pain has been shown to be associated with significantly worse health outcomes, as well as with depression [20].

#### Assessment of depression

The Center for Epidemiological Study of Depression (CES-D) scale was used to measure depression scores in patients, as it has demonstrated acceptable reliability amongst various patient groups [21]. The 20 item, self-reported questionnaire uses a four-point Likert scale to measure depressive symptoms, with scores ranging from 0 (lowest) to 60 (highest). Depression scores were divided into 3 levels based on a previous literature: no depression (level-1, CES-D < 15); mild depression (level-2, CES-D between 16 and 26); and moderate to severe (level-3, CES-D > 27) depression [3].

### Statistical analysis

Statistical analysis was performed using SPSS (Version 22.0. Armonk, NY: IBM Corp). Data was reported as mean ± SE or as percentages. One-way repeated measure analysis of variance (ANOVA) was performed to compare the pain intensity longitudinally across assessment time points. Pain severity was divided into 2 categories based on the NRS scores: no pain to moderate pain (score of 0–6), and severe pain (score of 7–10). The rate of pain (calculated as the number of patients with pain divided by the total number patients) and the percentage of patients with pain (calculated by multiplying the rate by 100) was calculated across assessment time points. One-way repeated measure analysis of variance (ANOVA) was performed to compare the depression scores longitudinally across assessment time points. Independent T-test analysis was performed to compare the CES-D scores at pre-operative, hospitalization, and 10 days post-discharge time points, between patients with no pain-to-moderate pain (NRS score of 0–6) and severe pain (NRS score of 7–10) post surgery.

A univariate logistic regression was performed to examine the association between pain intensity category (severe pain or no pain-moderate pain) and the following variables: pre-operative CES-D, hospitalization CES-D, CES-D 10 day post-discharge, age, sex, diabetes, ejection fraction, BMI, and smoking status. Multivariable logistic regressions were performed forcing all covariates with statistical significance of < 0.175 into the model. Backward variable elimination was used to develop the regression model. Those variables with a statistically significant level of < 0.1 were retained in the final model. In addition, the differences in depression scores between patients with severe pain and patients with no pain-moderate pain were examined using an independent-samples t-test. A *p*-value of < 0.05 was considered significant.

A 2-way repeated measure ANOVA was performed to assess levels of WBC longitudinally, comparing the 2 pain categories: no pain-moderate pain (NRS of 0–6) and severe pain (NRS 7–10) A *p*-value of < 0.05 was considered significant. The association between pain scores, CES-D scores, and WBC levels were assessed at pre-operative and post-operative time points using the Pearson correlation coefficient.

## Results

A total of 98 patients were included in this study. The mean age was 61 years old and there were 25% female patients. In this patient’s cohort, 36% of the patients were diagnosed with diabetes and the average BMI was 25.5 kg/m^2.^ 70% of the patients had preserved ejection fraction (left ventricle EF higher than 50%) and 40% were active smokers. Baseline characteristics in patients with No pain- Moderate pain and patients with severe pain are presented in Table 1.

**Table 1:** Baseline characteristics in patients with no pain- moderate pain compared to patients severe pain

	**No pain- Moderate pain (NRS 0–6)** (***n*** = 55)	**Severe Pain** **(NRS 7–10)** (***n*** = 43)	***p***-value
Age (years)	62.4 ± 8.6	58.8 ± 9.7	0.63
BMI (kg/m^2^)	27.5 ± 4.0	29.44 ± 4.2	0.30
Sex (female) % (n)	94.6% (*n* = 53)	70.5% (*n* = 31)	0.01
Diabetes % (n)	30.4% (*n* = 17)	45.5% (*n* = 20)	0.21
EF < 50% (n)	37.5% (*n* = 21)	22.7% (*n* = 10)	0.11
Non Smoking % (n)	32.1% (*n* = 18)	45.5% (*n* = 20)	0.17

**Fig. 1:**
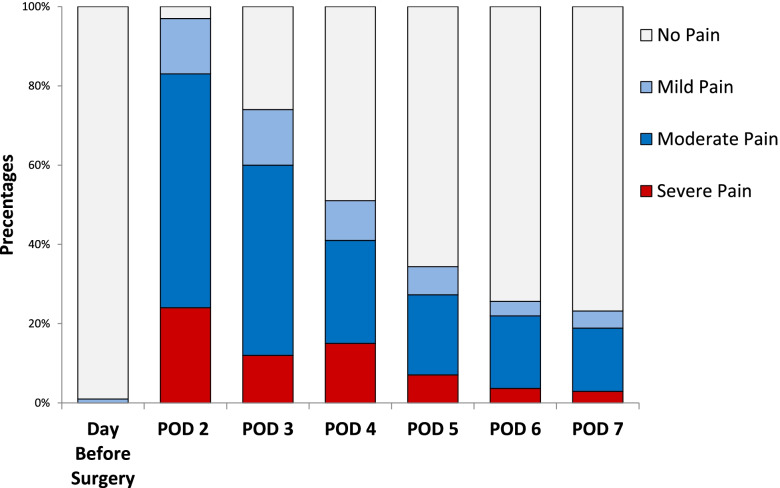
Perioperative percentage of patients with severe, moderate, and mild pain intensity. POD, post-operative day

Pain intensity increased significantly during hospitalization from pre-operative levels, peaking at post-operative day (POD) 2 (*p* < 0.0001). Pain intensity gradually decreased during the first week postoperatively 7 days of follow-up, but remained higher than the baseline levels (*p* < 0.0001). Although the percentage of patients with pain decreased gradually during the first week, the percentages of patients reporting moderate (NRS 4–6), and severe (NRS 7–10) pain remained high, with 83% of patients reporting moderate-severe pain on POD-2, 60% on POD-3, 41% on post-operative day- 4, 27% on post-operative day-5, 22% on post-operative day-6, and 19% post-operative day-7 (Fig. 1).

**Fig. 2:**
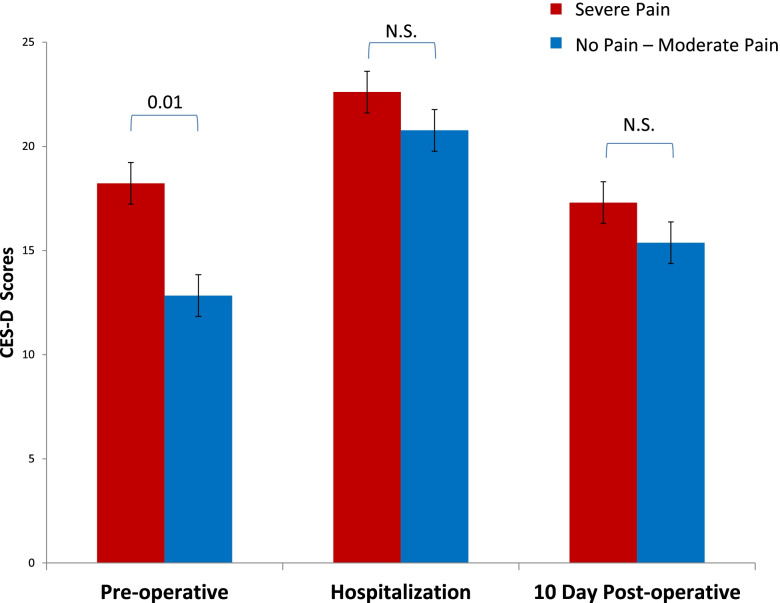
CES-D scores at preoperative, hospitalization, and 10 days post-operative time points in post-surgery patients with severe pain versus patients with no pain to moderate pain severity. Red bars represent severe pain category. Blue bars represent no pain to moderate pain intensity category. Y axis represents the Center for Epidemiological Study of Depression (CES-D) scores

Significantly higher pre-operatively CES-D scores were found in patients with severe (NRS 7–10) post-operative pain compared to patients with no pain to moderate pain (NRS 0–6) (Fig. 2) ([mean (CI)) 18.23 ± 1.80 (95% CI 14.59 – 21.87) vs 12.84 ± 1.22 ( 95% CI 10.40–15.28), *p* = 0.01). There was a trend of higher CES-D scores in patients with severe pain compared to patients with no pain-moderate pain during hospitalization (22.60 ± 1.46 (95%CI 19.65–25.56) vs. 20.77 ± 1.36 (95% CI 18.05–23.49) and 10-days post discharge (17.30 ± 1.72 (95% CI 13.83–20.77) vs. 15.38 ± 1.27 (95% CI 12.84–17.92), but the levels did not reach significance.

A Pearson correlation indicates that pre-operative CES-D scores was weakly correlated with maximum pain intensity (*r* = 0.2, *p* = 0.049). Hospitalization and 10 days post-discharge CES-D scores were also weakly correlated with maximum white blood cell count perioperatively (*r* = 0.229, *p* = 0.022 and *r* = 0.213, *p* = 0.035, respectively).

**Fig. 3:**
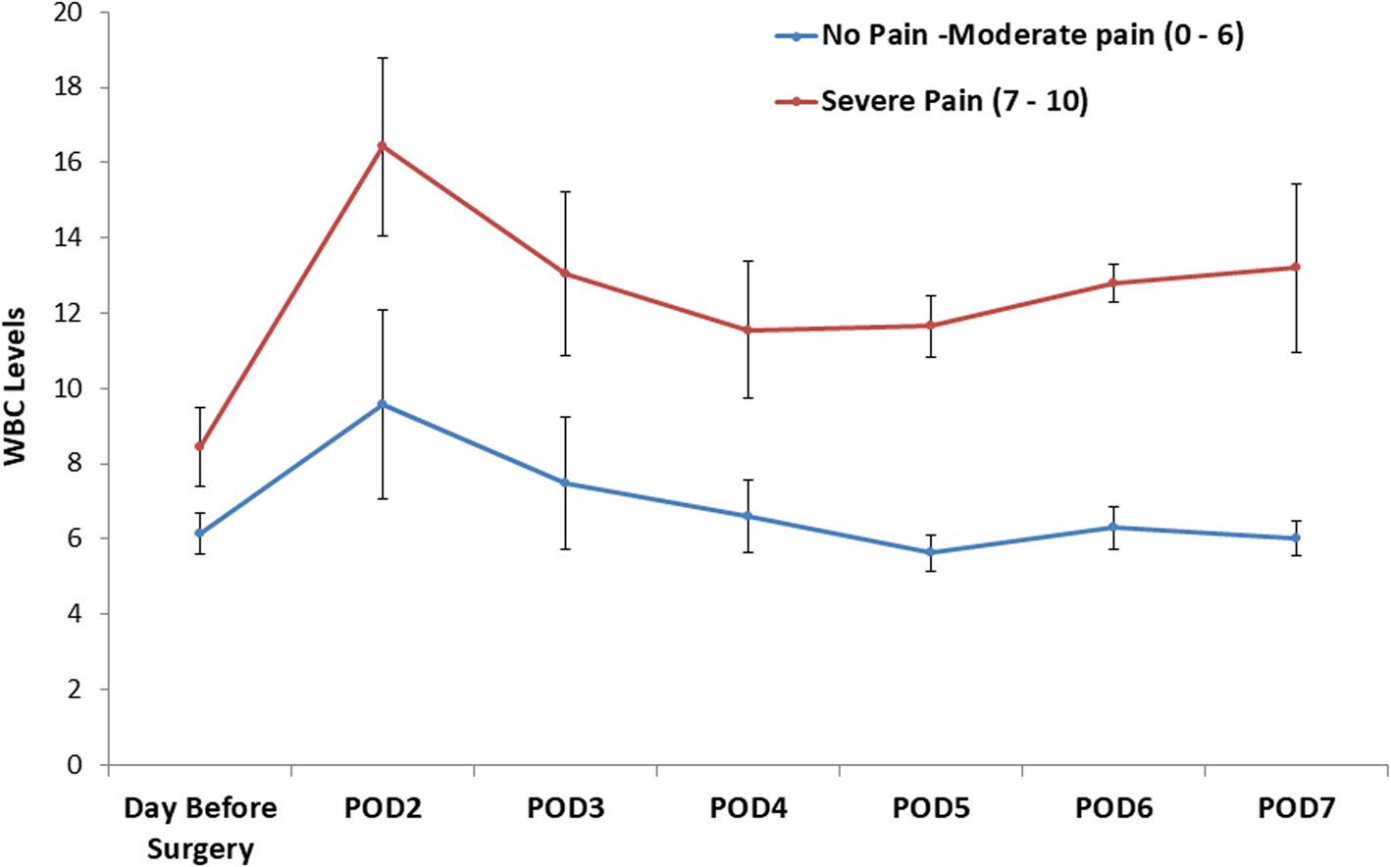
WBC levels during perioperative period. Red line represents severe pain category. Blue line represents no pain to moderate pain categories. Y axis represents white blood Cells levels (WBC). POD, post-operative day

A 2-way repeated measure ANOVA revealed that patients with severe pain (NRS 7–10) had significantly higher levels of WBC compared to patients with no to moderate pain (NRS 0–6), (*p* = 0.01) (Fig. 3). In both groups, the levels of WBC increased from pre-operative levels and peaked at day 2 post-operatively, followed by a gradual decrease in WBC levels.

A univariate analysis revealed that pre-operative CES-D, female sex, and high BMI were significant predictors of pain intensity during hospitalization (Table 2). In a multivariate regression, pre-operative CES-D, female sex, reduced left ventricle EF (< 50%), BMI, and smoking were associated with increased maximal pain scores (Nagelkerke *R*^*2*^ = 0.40,χ^2^ = 35.1 *p* < 0.001).

**Table 2:** Univariable and multivariable analysis of independent predictors of post-operative pain intensity

	**Univariable Analysis**		**Multivariable Analysis**	
**Parameter**	**OR (95%CI)**	***P***-value	**OR (95%CI)**	***P*-value**
Age	0.958 (0.916–1.003)	0.067	–––-	–––-
Sex	7.409 (1.957–28.049)	0.003	13.733 (2.676–70.485)	0.002
BMI	1.114 (1.008–1.232)	0.034	1.148(1.022–1.291)	0.020
Pre-op CES-D	1.050 (1.009 – 1.093)	0.016	1.059 (1.008 – 1.113)	0.023
Hospitalization CES-D	1.019 (.979–1.061)	0.360	–––-	–––-
10 Day Post-Discharge CES-D	1.019 (.980–1.059)	0.355	–––-	–––-
Diabetes	0.523 (0.23–1.191)	0.123	–––-	–––-
Ejection Fraction	2.050 (0.839–4.961)	0.116	4.259 (1.388–13.068)	0.011
Non Smoking	0.568 (0.251–1.286)	0.175	0.316 (0.111–0.897)	0.030

## Discussion

The main finding of the study is that patients with severe post-surgical pain had significantly higher depression scores pre-operatively than patients with no pain to moderate pain.

In our study, predictors of post-cardiac surgery pain intensity were pre-operative CES-D scores, smoking, high BMI, female sex, and ejection fraction (< 50%). The majority of these factors are modifiable. Studies have reported that cardiac surgery patients pain is more prevalent in depressive symptoms [6, 7]. Since uncontrolled pain post-cardiac surgery increases morbidity risk, preoperative depression screening may identify which patients require increased care following surgery [15, 17]. In addition, it is possible that pre-operative treatments of depression may reduce risk of developing severe pain post-cardiac surgery. Furthermore, interventions aiming at lifestyle modification, such as smoking cessation during the pre-operative period that were shown to be beneficial for many clinical outcomes [22, 23], may also be beneficial for pain management. In addition, exercise training was reported have beneficial effects on depression [24]. Patients who exercised prior to the surgery may experience less depression symptoms and lower pain intensity.

In our patient population, pain peaked early in the post-operative period, which is consistent with current literature [8, 25]. In fact, uncontrolled pain post-cardiac surgery increases morbidity risk [15, 17]. Our findings that women experienced higher pain intensity is consistent with previous literature examining post-surgical pain, with previous studies observing a higher rate of pain in women [26, 27]. Studies have also noted a greater frequency of persistent pain at 12 months post-cardiac surgery in women compared to men [28]. Our study observed no significant association between CES-D scores during hospitalization and maximum pain intensity. However, we found a significant association between maximum pain intensity during hospitalization and pre-operative CES-D levels. One reason for the lack of significant association between pain intensity and CES-D scores during hospitalization may be due to the use of multiple pain medications being used throughout the hospitalization period, which may have affected each patient’s subjective pain scores. Interestingly, we found that prior to surgery, 1% of our cohort experienced pre-operative pain. Pre-operative pain is common, with 38% of cardiac surgery patients reporting pain prior to surgery [8]. Further research should investigate whether treatment of pain prior to surgery may affect postoperative outcomes, including subjective pain symptoms, and whether pre-operative pain is linked with greater rates of reoperation. It is important to note that our study included a small sample size. Future larger studies are required to confirm our findings. With anti-depressants typically taking a few weeks for clinical effects to become apparent, pre-operative treatment with a selective serotonin reuptake inhibitor (SSRI) or selective norepinephrine reuptake inhibitor (SNRI) with the addition of anti-inflammatory medications post-operatively may be an option to be further investigated.

Pain and depression have been noted to have significant comorbidity and both involve similar neural networks and similar neurotransmitter systems that influence neuroplasticity in the brain [10, 11]. Imaging studies have been able to elicit a large overlap in brain regions implicated in both disorders, such as the prefrontal cortex, anterior cingulate cortex, nucleus accumbens, and amygdala [12, 13]. Recent studies have also been able to identify key neural circuits in comorbid depressive symptoms and chronic pain, with the lateral habenula leading to an activation of GABAergic neurons in the dorsal raphe nucleus, via the central nucleus of the amygdala [29].

Post-surgical pain may also be linked not only to depressive symptoms, but also to the trauma associated with the procedure and the elevated white blood cell (WBC) levels induced by the surgery [30, 31]. In this study, we evaluated the levels of WBC, since their levels may be related to the trauma associated with the surgical procedure [30, 31]. Our study found a significant association between WBC levels and pain intensity levels throughout the hospitalization period. However. Our findings of an association between CES-D scores and increased WBC levels are consistent with a recent meta-analysis which noted that major depressive disorder is associated with overall leukocytosis [32]. Future studies should examine whether patients in this population may benefit from a multi-pronged approach targeting pain and depression.

## Conclusion

Pain intensity significantly increased post-cardiac surgery, and is associated with depressive symptoms, female sex, cardiac function, BMI, and smoking. These factors may serve as a basis for identification and intervention to help prevent the transition from acute pain to chronic pain. Moreover, physicians should anticipate higher levels of pain in patients with higher CES-D scores. The high depressive scores found post-operatively highlight the need to establish depression screening as part of the pre-operative patient management. Future research should investigate the effectiveness of post-cardiac surgery pain treatments and determine whether perioperative treatment with antidepressants medications can affect patient outcomes.

## Data Availability

The data is available upon request from the corresponding author.

## Abbreviations

CES-D: Center for Epidemiological Study of Depression
WBC: White blood cells
BMI: Body mass index
CABG: Coronary artery bypass grafting
EF: Ejection fraction
NRS: Numerical Rating System
ANOVA: Analysis of variance
POD: Post-operative day
SSRI: A selective serotonin reuptake inhibitor
SNRI: Selective norepinephrine reuptake inhibitor
